# Quality of Life and Out-of-Pocket Expenditures for Sickle Cell Disease Patients in Saudi Arabia: A Single-Center Study

**DOI:** 10.3390/healthcare12212146

**Published:** 2024-10-29

**Authors:** Yazed AlRuthia, Rayan B. Alanazi, Sultan F. Alotaibi, Miteb Alanazi

**Affiliations:** 1 Department of Clinical Pharmacy, College of Pharmacy, King Saud University, P.O. Box 2454, Riyadh 11451, Saudi Arabia; 2 Department of Pharmacy, King Khalid University Hospital, P.O. Box 7805, Riyadh 11472, Saudi Arabia

**Keywords:** sickle cell disease, out-of-pocket expenditures, blood transfusion, health-related quality of life, electronic health record data, Saudi Arabia

## Abstract

**Background:** Sickle cell anemia (SCD) is a relatively uncommon health condition in many countries, but it is prevalent in Saudi Arabia mainly due to the high incidence of consanguineous marriages. Regrettably, there are elevated rates of vaso-occlusive crises (VOCs) and blood transfusions, leading to poor quality of life and significant financial strain. **Objective(s):** This study aimed to assess the frequency of blood transfusions, out-of-pocket expenditures (OOPEs), and health-related quality of life (HRQoL) in SCD patients. **Methods:** This was a questionnaire-based cross-sectional study that involved SCD patients at a university-affiliated tertiary care center in Riyadh, Saudi Arabia. The patients’ medical and sociodemographic characteristics were obtained from the electronic medical records. Data on HRQoL and OOPEs were collected through a questionnaire-based interview. To present the baseline characteristics, descriptive statistics such as mean, standard deviation, frequency, and percentage were used. In addition, various statistical tests, including the Chi-Square test, Student *t*-test, one-way ANOVA, and multiple linear regression, were performed. **Results:** One hundred and eighteen patients consented to participate and were included in the analysis. Almost 53% of the patients were females. The mean age of the sample was 31 years, while the age-adjusted quality-adjusted life years (QALYs) was 24.33 years (*p*-value < 0.0001). Most patients (83.05%) reside in Riyadh with a monthly family income of less than USD 2666.67 (75.42%). Monthly OOPEs were, on average, USD 650.69 ± 1853.96, and one-third of the adult patients reported income loss due to illness, further exacerbating their financial strain. High frequency of blood transfusion (β = −0.0564, *p*-value = 0.0066) and higher number of comorbidities (β = −0.10367, *p*-value = 0.0244) were negatively associated with the HRQoL among adult patients. On the other hand, adult patients with higher levels of education had better HRQoL (β = 0.05378, *p*-value = 0.0377). **Conclusions:** The findings of this study highlight the negative impact of SCD on patients’ HRQoL and financial well-being. This underscores the urgent need for comprehensive systemic approaches to address the challenges posed by SCD in Saudi Arabia.

## 1. Introduction

Sickle cell disease (SCD) encompasses a range of hematological disorders that alter the typical shape of red blood cells, leading to impaired blood flow, severe pain, tissue damage, anemia, and other serious complications [1]. The most common and severe form of SCD is sickle cell anemia (SCA), which is just one of several types with varying degrees of severity [2]. There are approximately 250 million carriers of the SCD gene worldwide, with increasing numbers in countries like Saudi Arabia, Cameroon, Gabon, Ghana, Nigeria, and India [3]. From 2000 to 2021, the number of individuals living with SCD is estimated to have grown by 41% to reach 7.74 million [3]. In 2021 alone, over half a million infants were born with SCD, particularly in sub-Saharan African countries [3,4]. In Saudi Arabia, an estimated 2.3% of the population has SCD, and about 3.1% are carriers of the SCD trait, mainly due to high rates of consanguineous marriages [5,6]. This prevalence contrasts with the much lower rates of 0.0005% in Europe and approximately 0.0004% in the United States [7,8,9].

The economic burden of sickle cell disease (SCD) is significant due to its severe complications and the necessity for timely medical care to alleviate pain and improve patients’ quality of life [10,11,12]. In the United States, the estimated lifetime medical costs associated with SCD for patients under 65 years of age, based on commercial healthcare claims data, exceed $1.5 million, with lifetime out-of-pocket expenditures (OOPEs) estimated to be over $42,000. These costs are more than 900% higher than those of non-SCD patient populations [10]. In Nigeria, where the prevalence rate ranges between 2% and 3%, the median annual medical expenditures for SCD are $4620 per person, double the country’s GDP per capita [11]. In Italy, the mean annual direct medical cost for SCD patients with at least one hospitalization within a year was estimated to be €7918 per patient based on retrospective analysis of administrative databases [13]. These figures underscore the considerable economic impact of SCD on patients and healthcare systems. Despite the relatively high prevalence of SCD in Saudi Arabia, there are no estimated direct or indirect costs for patients with SCD. However, a single-center retrospective study revealed high hospital readmissions and emergency department visits among SCD patients, indicating the country’s unmeasured economic burden of the disease [14].

The many complications of sickle cell disease (SCD), including vaso-occlusive crises (VOCs), organ damage, frequent hospitalizations, and emergency department visits, significantly reduce the health-related quality of life (HRQoL) for patients [15,16,17,18]. A longitudinal cohort study conducted in Virginia, United States, between July 2002 and August 2004 found that patients with SCD had a similar HRQoL to those with end-stage renal disease (ESRD) [15]. A simulation study in the United States estimated that there are around 1950 newborns with SCD annually, and the quality-adjusted life expectancy for SCD patients was reported to be 33 years, compared to 67 years for matched non-SCD patients. It was also estimated that patients with SCD may experience a $700,000 loss in income over their lifetime compared to matched non-SCD patients [19]. In Saudi Arabia, several studies have shown that patients with SCD have diminished HRQoL. However, none have reported age-adjusted quality-adjusted life years (QALYs) since the publication of the EuroQol-5-Domain-5-Levels (EQ-5D-5L) value set for the Kingdom of Saudi Arabia [5,20,21]. Therefore, this study aimed to investigate the impact of SCD on patients’ HRQoL, report age-adjusted QALYs, and assess the economic impact of SCD by measuring SCD-related out-of-pocket expenditures (OOPEs) on patients and their families.

## 2. Methods

### 2.1. Study Design and Patient Population

This study was conducted through interviews and was cross-sectional. Patients with sickle cell disease (SCD) were identified from the electronic medical records (EMRs) of a university-affiliated tertiary care center in Riyadh, Saudi Arabia. The hospital is a large institution with over 900 staffed beds and offers various specialized medical services, including hematology. Patients with SCD, as indicated in their EMRs, who had been diagnosed with the condition for at least one year to estimate the annual financial impact of SCD on patients and their families were included in the study. Those with a disease duration of less than one year and those who had only one medical encounter in the past 12 months and were not scheduled for further follow-up visits were excluded. Additionally, patients with hematological or non-hematological malignancies were also excluded. Convenience sampling was employed, and data collection commenced on 21 November 2023, concluding on 24 March 2024.

### 2.2. Study Tools and Variables

The patients’ demographic and medical characteristics, such as age, gender, age at diagnosis, type of sickle cell disease, prescribed medications, comorbidities, and frequency of blood transfusions, were extracted from the electronic medical records (EMRs). To evaluate the patient’s HRQoL, we utilized the Arabic version of the EQ-5D-5L and the recently published value set for the Saudi Arabian population [21]. Out-of-pocket expenses (OOPEs) were evaluated using the Arabic version of the Costs for Patients Questionnaire (CoPaQ), which covers costs related to illness, including expenses for medical visits, prescription and non-prescription medications, travel and accommodations for medical consultations, and supplemental health insurance [22,23]. Additionally, we collected information on patients’ family income, educational level, marital status, healthcare coverage, region of residence, and educational level. During waiting times at the outpatient clinics, two pharmacy interns interviewed patients or their caregivers (e.g., mothers or fathers) if the patients were under 18. Interviews regarding HRQoL and financial stress levels were only conducted with adult patients aged 18 years and above, as the Arabic EQ-5D-5L is validated only for adults in this age group [21].

### 2.3. Statistical Analysis

The study utilized descriptive statistics, such as means, standard deviations, medians, minimums, maximums, frequencies, and percentages, to characterize the baseline attributes, quality of life domains (including mobility, self-care, usual activities, pain/discomfort, and anxiety/depression), and out-of-pocket expenses (OOPEs). Statistical significance between male and female participants for Visual Analog Scale (VAS) scores, age-adjusted QALYs, and financial stress levels was assessed using Chi-Square and Student’s *t*-test. Additionally, multiple linear regression was performed to explore the relationship between EQ-5D utility scores and the frequency of blood transfusion, number of comorbidities, education, gender, and age. The minimum sample size was determined to be 90 patients using G-Power^®^ software version 3.1 with α = 0.05, β = 0.05, 95% power, and a medium effect size (Cohen’s d of 0.6) for a two-tailed *t*-test. All statistical analyses were conducted using SAS^®^ version 9.4 (SAS Institute, Cary, NC, USA).

### 2.4. Ethical Considerations

The pharmacy interns requested that patients or their caregivers sign a written consent form explaining the study’s purpose and their right to withdraw from the interview at any time without facing any penalty. Following the signing of the consent form, the interview typically lasted around 15 min. No personal identifiers, such as name, national ID, address, and medical record numbers, were gathered. All the collected data were anonymized and securely stored, with access restricted to only the primary investigator. The study adhered to the ethical principles outlined in the Helsinki Declaration [24].

## 3. Results

Out of 160 eligible patients meeting the inclusion criteria, 118 consented to participate in the study. All participating patients had the Hb SS SCD genotype. Among the participants, 53% were female, and nearly 84% were adults (age ≥ 18 years). Furthermore, 76.27% of the participants had a high school diploma or college degree. Moreover, 83% of the participants resided in Riyadh, and approximately 69% were single. A majority (61.86%) of the participants were diagnosed before age two. Almost 90% of the participants belonged to families with a monthly income of less than USD 4000. Most patients did not have other chronic health conditions. However, conditions such as cardiovascular disease, asthma, diabetes, osteoarthritis, stroke, and hypertension were present in less than 9% of the participants, as detailed in Table 1. Additionally, a vast majority of the patients (91.92%) were treated with hydroxyurea, while only 10.10% received opioid analgesics. Most patients were treated with one or two prescription medications (72.03%). For most patients, regular monthly blood transfusions were not received (90.67%), and only 3.39% of patients had supplemental private health insurance, as shown in Table 1.

**Figure 1:**
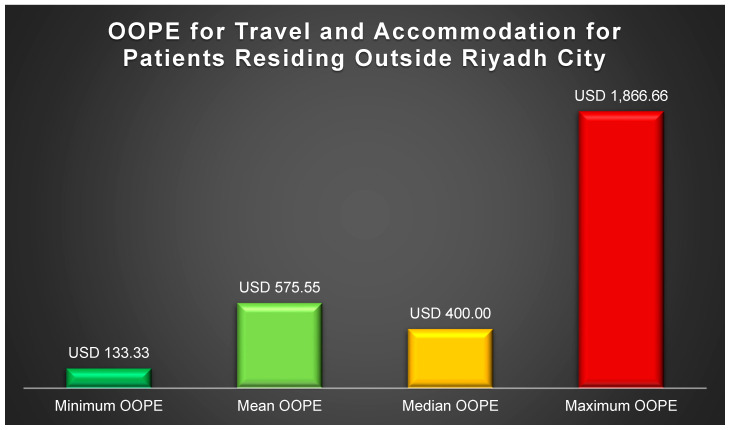
Out-of-pocket expenditures (OOPE) for travel and accommodation among patients outside Riyadh city.

**Figure 2:**
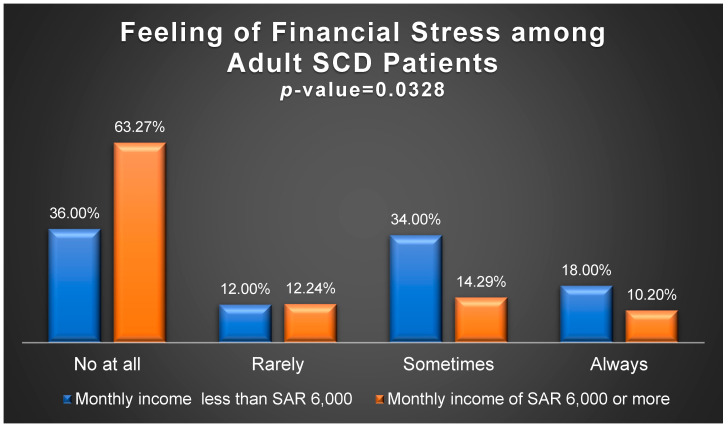
Financial stress feeling among adult patients stratified by individual monthly income.

**Figure 3:**
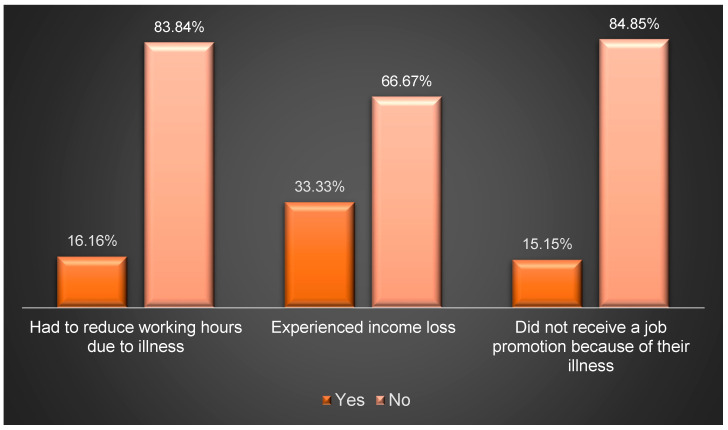
Impact of SCD on adult patients’ careers.

The mean monthly out-of-pocket expenses (OOPEs) for healthcare services, comprising medical visits, prescription drugs, lab tests, and imaging studies, amounted to USD 523.28, representing 80.42% of the total monthly OOPEs, as outlined in Table 2. Meanwhile, Figure 1 illustrates the range of OOPEs for travel and accommodation for patients outside Riyadh. Given that over 50% of the participants come from families with a monthly income of less than USD 1600, this figure served as a benchmark for comparing the proportions of participants experiencing financial stress. As indicated in Figure 2, nearly 63.27% of adult participants from families earning USD 1600 or more per month reported no financial stress due to SCD, compared to 36% of participants from families earning less than USD 1600 (*p*-value = 0.0328). Furthermore, 24.49% of participants from families earning USD 1600 or more per month felt financial pressure occasionally or regularly, in contrast to 52% among families earning less than USD 1600 (*p*-value = 0.0328). Additionally, Figure 3 depicts the impact of SCD on the careers of adult patients (≥18 yrs.). At least two-thirds of the adult participants had to reduce working hours, experienced income loss, or did not receive a job promotion due to their illness, as highlighted in Figure 3.

**Figure 4:**
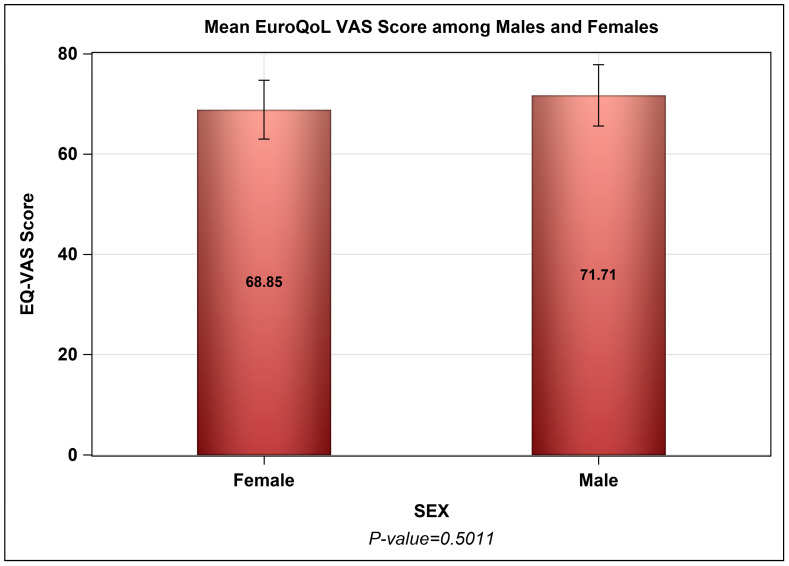
Mean scores of the EuroQol-5D-5L Visual Analog Scale (VAS) among male and female patients.

**Figure 5:**
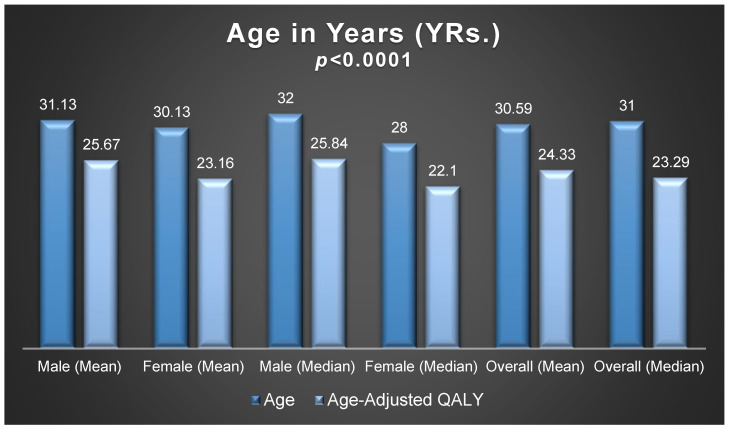
Age and age-adjusted QALYs among male and female patients.

The vast majority of adult patients (≥18 yrs.) reported either “no” or “slight” difficulties in mobility (74.75%), self-care (86.87%), usual activities (69.69%), pain/discomfort (61.86%), and anxiety/depression (61.02%), as depicted in Table 3. The mean EuroQol-visual analog scale (EQ-VAS) score, used to evaluate general well-being, was 70.18 ± 20.93, with no discernible difference between male and female participants, as seen in Figure 4. The overall mean utility score was 0.793, resulting in a mean age-adjusted QALY of 24.33 ± 10.25 years, significantly lower than the mean age of the participants (30.59 ± 9.11 yrs., *p*-value < 0.0001), which also holds true across gender, illustrated in Figure 5. Among the 99 adult patients, a high frequency of blood transfusion (β = −0.0564, *p*-value = 0.0066) and a higher number of comorbidities (β = −0.10367, *p*-value = 0.0244) were found to be negatively associated with EQ-5D utility of life scores. On the other hand, adult patients with higher levels of education exhibited better quality of life (β = 0.05378, *p*-value = 0.0377) as shown in Table 4.

## 4. Discussion

Saudi Arabia has a relatively high prevalence rate of SCD in comparison to the global prevalence rate [3,14]. However, despite its high prevalence, no national or local studies about the social and economic impact of SCD in the Kingdom were conducted compared to other countries with significantly lower prevalence rates in Europe, Brazil, and the United States [10,12,16,17]. Therefore, there is a need to explore the social and economic impact of SCD on the Saudi Arabian population, which was the aim of this study. The results of this study revealed many facts that were largely unreported in Saudi Arabia. Most patients belonged to families with a monthly family income of less than SAR 14,823 (USD 3952.8), less than the average household monthly income among Saudi nationals based on the 2018 Household Income and Expenditure Survey [25]. This is in line with most of the published studies that explored the sociodemographic characteristics of SCD patients. These studies have consistently found that most patients come from low-income families and countries [4,11,26]. About 50% of the patients did receive at least one red blood cell transfusion within a year, which is considered the mainstay of therapy for patients with SCD, but could be associated with severe complications and should be tailored to patient needs [27,28]. This rate is lower than reported for hospitalized SCD patients in the United States or the MENA region, with blood transfusion rates of over 70% [29,30]. Most patients were treated with hydroxyurea, which has proven its value in reducing the incidence of VOCs among Saudi patients with SCD [31]. Moreover, the rate of opioid analgesic utilization among the patients in this study was low and is consistent with most of the published studies about the use of opioid analgesics for the management of VOCs, which are mostly limited to hospitalized patients [32].

The out-of-pocket expenditures (OOPEs) incurred by families with members affected by sickle cell disease (SCD) were substantial, representing between 24% and 41% of the monthly income for families earning less than SAR 10,000 ($2666.67), and accounted for 75% of the patients. Furthermore, the financial strain SCD patients and their caregivers felt was significantly higher among those earning less than SAR 6000 ($1600), who made up more than 50% of the patients. This presents a significant welfare issue, as reported in a study that examined the impact of health-related OOPEs among Saudi families using nationally representative data [33]. This finding aligns with other studies that reported noteworthy OOPEs among SCD patients in low- and high-income countries [18,19,26].

Patients outside Riyadh reported varying OOPEs for travel and accommodation, highlighting the challenges of accessing specialized care in their geographical areas [34,35]. Consequently, access to specialized care for families with SCD-affected patients in their respective regions should be enhanced. Additionally, at least 15% of adult SCD patients experienced income loss, had to reduce their working hours, or missed out on job promotions due to their illness. This is consistent with prevailing evidence that indicates higher rates of absenteeism and employment disruptions among SCD patients [19,36,37]. However, the percentage of individuals affected by these challenges in Saudi Arabia and other Arabian Gulf countries may be lower due to the social support provided by their governments and families [38].

Most adult patients with sickle cell disease (SCD) reported no issues or minimal problems across the five domains assessed by the EuroQol-5D-5L. However, their mean utility score was 0.792, resulting in an average age-adjusted QALYs of 24.33 years, which is notably lower than the mean age of 30.59 years. This underscores the profound impact of SCD on the HRQoL of patients, resulting in an approximate six-year loss. These findings align with previous studies emphasizing the substantial negative impact of SCD on the HRQoL of patients [18]. Notably, the disutility associated with SCD in this study is comparatively lower than in most published studies, which have reported higher disutility scores among SCD patients [15,19]. Nevertheless, these findings are consistent with a real-world cross-sectional study that reported similar disutility scores among SCD patients not experiencing vaso-occlusive crises (VOCs) [39]. Additionally, many previous studies have been based on cohort simulations or have focused on assessing HRQoL among hospitalized patients with VOCs [16,17,18,39]. Apart from the frequency of blood transfusions, which was linked to poorer HRQoL, a higher number of comorbidities was associated with lower HRQoL, while a higher level of education was linked to improved HRQoL. These findings align with prior research indicating a positive correlation between educational attainment and HRQoL [40] and a negative association between comorbidity burden and HRQoL [41].

Finally, although this study is the first to estimate the OOPEs, HRQoL, and utility of life among SCD patients, it has multiple limitations that must be acknowledged. First, this is a single-center study with a small sample size, limiting the findings’ generalizability. Secondly, the study only included patients with the HbSS genotype, most of whom were from the Riyadh region. This further limits the generalizability of the findings, considering that most SCD patients in Saudi Arabia reside in the southern and eastern regions. Thirdly, the study included non-hospitalized patients, so it did not capture the impact of SCD complications, such as VOCs, on HRQoL. Moreover, about 26.3% of the patients did not consent to participate in the study, which may have influenced the study’s findings.

## 5. Conclusions

The findings of this study shed light on the significant social and economic impact of sickle cell disease (SCD) from the perspective of patients in Saudi Arabia for the first time. Future studies with more representative data are essential to validate these findings and evaluate the burden of illness among patients with different genotypes of SCD across various regions. Additionally, estimating the direct medical costs of SCD using nationally representative data is crucial to assessing the impact of newly approved gene therapies for SCD on both clinical and financial outcomes.

## Figures and Tables

**Table 1:** Patient baseline characteristics (*n* = 118).

Characteristic	Frequency (%)
**Gender**	
Male	56 (47.46)
Female	62 (52.54)
**Age group**	
0–5 yrs.	1 (0.85)
6–11 yrs.	7 (5.93)
12–17 yrs.	11 (9.32)
18–23 yrs.	27 (22.88)
24–29 yrs.	22 (18.64)
30–35 yrs.	20 (16.95)
36–41 yrs.	18 (15.25)
42–47 yrs.	7 (5.93)
48–53 yrs.	5 (4.24)
**Education**	
Pre-school and nursery	2 (1.69)
Elementary school	11 (9.32)
Intermediate school	15 (12.71)
High school	46 (38.98)
College degree	44 (37.29)
**Region of residence**	
Riyadh	98 (83.05)
Makkah	1 (0.85)
Jizan	9 (7.63)
Tabuk	2 (1.69)
Eastern region	3 (2.54)
Hail	2 (1.69)
Albaha	3 (2.54)
**Marital Status**	
Single	81 (68.64)
Married	35 (29.66)
Divorced	2 (1.69)
**Age at diagnosis**	
0–2 yrs.	73 (61.86)
2–4 yrs.	23 (19.49)
4–6 yrs.	14 (11.86)
6–8 yrs.	4 (3.39)
8–10 yrs.	2 (1.69)
10–12 yrs.	1 (0.85)
>12 yrs.	1 (0.85)
**Family monthly income in United States Dollars (USD)**	
<800 USD	28 (23.73)
800–1600 USD	34 (28.81)
1600–2666.67 USD	27 (22.88)
2666.67–4000 USD	17 (14.41)
4000–5333.33 USD	10 (8.47)
>5333.33 USD	2 (1.69)
**Other comorbidities**	
Cardiovascular disease	3 (4.24)
Asthma	8 (6.78)
Diabetes	3 (2.54)
Osteoarthritis	10 (8.47)
Stroke	2 (1.69)
Hypertension	3 (2.54)
**Number of prescription medications**	
1–2 Medications	85 (72.03)
3–4 Medications	33 (27.97)
**Frequency of simple blood transfusion**	
Regularly (i.e., Every month)	11 (9.32)
Sometimes (i.e., Once to twice a year)	49 (41.53)
Rarely (i.e., Every two to three years)	7 (5.93)
No blood transfusion	51 (43.22)
**Was there a delay in blood transfusion when you needed it?**	
No	106 (89.83)
Yes	12 (10.17)
**Healthcare services are paid by:**	
Employer-based private health insurance	4 (3.39)
Public healthcare coverage	114 (96.61)

**Table 2:** Monthly patient out-of-pocket expenditures (OOPEs).

Component of Out-of-Pocket Expenditures	Mean ± SD
Child care	USD 88.59 ± 241.22
Healthcare services (i.e., medical visits, prescription drugs, lab tests, imaging studies)	USD 523.27 ± 1822.97
Other costs (e.g., car parking, special meals, etc., …)	USD 38.82 ± 134.12
**Total cost (USD)**	USD 650.69 ± 1853.96

**Table 3:** Quality of life of adult patients with SCD assessed by the EUROQol-5D-5L (*n* = 99).

Domain	Frequency (%)
**Mobility**	
No problems in walking about	57 (57.58)
Slight problems in walking about	17 (17.17)
Moderate problems in walking about	16 (16.16)
Severe problems in walking about	9 (9.09)
Unable to walk about	0 (0)
**Self-care**	
No problems washing or dressing	78 (78.79)
Slight problems washing or dressing	8 (8.08)
Moderate problems washing or dressing	12 (12.12)
Severe problems washing or dressing	1 (1.01)
Unable to wash or dress	0 (0)
**Usual activities**	
No problems doing usual activities	36 (36.36)
Slight problems doing usual activities	33 (33.33)
Moderate problems doing usual activities	21 (21.21)
Severe problems doing usual activities	6 (6.06)
Unable to do usual activities	3 (3.03)
**Pain/Discomfort**	
No pain or discomfort	31 (31.31)
Slight pain or discomfort	42 (42.42)
Moderate pain or discomfort	16 (16.16)
Severe pain or discomfort	7 (7.07)
Extreme pain or discomfort	3 (3.03)
**Anxiety/Depression**	
Not anxious or depressed	49 (49.49)
Slightly anxious or depressed	23 (23.23)
Moderately anxious or depressed	19 (19.19)
Severely anxious or depressed	6 (6.06)
Extremely anxious or depressed	2 (2.02)

**Table 4:** Multiple linear regression for the association between EQ-5D utility score and frequency of blood transfusion among adult patients (*n* = 99).

Variable	Β-Estimate	*p*-Value
Frequency of blood transfusion	−0.05645	0.0066 *
Number of comorbidities	−0.10367	0.0244 *
Age	0.00143	0.5570
Female versus male	−0.02195	0.6167
Education	0.05378	0.0377 *

* *p* < 0.05.

## Data Availability

The data are available upon reasonable request from the corresponding author.
